# Transcriptomic analysis provides insight into the genetic regulation of shade avoidance in *Aegilops tauschii*

**DOI:** 10.1186/s12870-023-04348-y

**Published:** 2023-06-23

**Authors:** Die Xie, Ming Hao, Laibin Zhao, Xue Chen, Xuejiao Chen, Bo Jiang, Shunzong Ning, Zhongwei Yuan, Lianquan Zhang, Kai Shu, Yijing Zhang, Dengcai Liu, Peipei Wu

**Affiliations:** 1 Triticeae Research Institute, Sichuan Agricultural University at Chengdu, Wenjiang, 611130 Sichuan China; 2 Henan Provincial Key Laboratory of Hybrid Wheat, School of Life Science and Technology, Henan Institute of Science and Technology, Xinxiang, 453003 China; 3 State Key Laboratory of Crop Gene Exploration and Utilization in Southwest China, Sichuan Agricultural University at Chengdu, Wenjiang, 611130 Sichuan China; 4 School of Ecology and Environment, Northwestern Polytechnical University, Xi’an, 710012 China; 5 State Key Laboratory of Genetic Engineering, Collaborative Innovation Center of Genetics and Development, Department of Biochemistry, Institute of Plant Biology, School of Life Sciences, Fudan University, Shanghai, 200438 China

**Keywords:** *Aegilops tauschii*, Wheat, Differentially expressed genes, Shade avoidance

## Abstract

**Background:**

Weeds are not only economically important but also fascinating models for studying the adaptation of species in human-mediated environments. *Aegilops tauschii* is the D-genome donor species of common wheat but is also a weed that influences wheat production. How shading stress caused by adjacent wheat plants affects *Ae. tauschii* growth is a fundamental scientific question but is also important in agriculture, such as for weed control and wheat breeding.

**Result:**

The present study indicated that shade avoidance is a strategy of *Ae. tauschii* in response to shading stress. *Ae. tauschii* plants exhibited growth increases in specific organs, such as stem and leaf elongation, to avoid shading. However, these changes were accompanied by sacrificing the growth of other parts of the plants, such as a reduction in tiller number. The two reverse phenotype responses seem to be formed by systemically regulating the expression of different genes. Fifty-six genes involved in the regulation of cell division and cell expansion were found to be downregulated, and one key upstream negative regulator (*RPK2*) of cell division was upregulated under shading stress. On the other hand, the upregulated genes under shading stress were mainly enriched in protein serine/threonine kinase activity and carbon metabolism, which are associated with cell enlargement, signal transduction and energy supply. The transcription factor *WRKY72* may be important in regulating genes in response to shading stress, which can be used as a prior candidate gene for further study on the genetic regulation of shade avoidance.

**Conclusions:**

This study sheds new light on the gene expression changes and molecular processes involved in the response and avoidance of *Ae. tauschii* to shading stress, which may aid more effective development of shading stress avoidance or cultivars in wheat and other crops in the future.

**Supplementary Information:**

The online version contains supplementary material available at 10.1186/s12870-023-04348-y.

## Background

The emergence of agriculture created habitats not only for intentionally cultivated plants (crops) but also for unwanted species (weeds) that adapted to exploit human-mediated environments. Agricultural weeds are among the great survivors of the plant kingdom, to persist and thrive in the face of human persecution or attempts to kill [1]. In evolution, weeds and crops may interact. For instance, all present-day cultivated varieties of Asian rice (*Oryza sativa*) were domesticated from the wild species *O. rufipogon* ~ 10 000 years ago [2, 3]. However, *de novo* weedy rice strains were also generated through domestication during the history of rice cultivation [4–6]. Furthermore, gene introgression from cultivated rice into weedy rice has led to the formation of a genetically and morphologically variable group, enhancing the adaptation of weedy strains to diverse anthropogenic environments [7].

Weeds can contribute to the evolution of polyploid crops. A well-known example is common wheat (*Triticum aestivum*, 2n = 6x = 42, AABBDD). This crop originated from the hybridization between cultivated wheat *T. turgidum* (AABB) and the weed *Aegilops tauschii* (DD) ~ 10,000 years ago [8, 9]. *T. turgidum* cultivation is still associated with weedy *Ae. tauschii* in Middle Eastern agroecosystems, which is thought to be the birthplace of common wheat [10]. The incorporation of the D-genome of *Ae. tauschii* is crucial for the success of bread wheat as the largest acreage crop in the world since it confers the potential to make diverse food products and a broad adaptability to diverse environments [11]. Natural introgression from other weedy species, such as wild emmer wheat, increases the genetic diversity of common wheat after origination [12]. Artificial introgression of genes from wild species into wheat has been widely exploited as a popular tool in modern breeding [13].

Wheat-*Ae. tauschii* is a fascinating model to study the growth competition of polyploid crops with progenitor species. *Ae. tauschii* is an invasive weed affecting wheat production, but controlling it is difficult due to its close evolutionary relationship with wheat. For example, in China, before the 1990s, this species was only sporadically observed in wheat fields in three provinces [14]. However, it escaped control management and quickly expanded to new habitats. By 2007, it occurred in eight provinces with a damage area of ~ 3.3 × 10^5^ ha [15]. Malignant weeds can cause up to 50–80% yield loss [16, 17]. To better control this weed, it is crucial to understand the adaptation mechanism of its competition with wheat.

Competition for sunlight in plants can be particularly fierce since photosynthesis is the only source of energy [18]. Shade avoidance and shade tolerance are two main strategies to adapt to competition for light changes caused by adjacent vegetation [19]. Here, the phenotypes and global gene transcription profiles of *Ae. tauschii* when grown alone and co-planted with wheat were compared. The picture that emerges is that *Ae. tauschii* exhibited phenotypic shade-avoidance responses. Correspondingly, gene transcription changed to adapt to competition. This study provides fundamental insight into the regulatory process underlying shade avoidance in *Ae. tauschii* and facilitates the identification of genes involved in shade-avoidance mechanisms.

## Materials and methods

### Plant materials and experimental setup

The common wheat variety Shumai969 (bred by Dengcai Liu, Triticeae Research Institute of Sichuan Agricultural University, China) [13] and four *Ae. tauschii* accessions, two from subspecies *tauschii* (AS71 and AS77) and two from subspecies *strangulata* (PI431599 and PI431602), were used in this study. AS77 was collected from the wheat fields of Hennan Province, China [14]. The remaining three were collected from the natural distribution areas of the species, AS71 from Xinjiang, China, PI431599 from Azerbaija, and PI431602 from Turkmenistan. AS71 and AS77 were formally identified by Chi Yen (the Triticeae Research Institute of Sichuan Agricultural University, China) and provided by Sichuan Agricultural University. PI431599 and PI431602 were formally identified by N. I. Vavilov (Institute of Plant Industry, Former Soviet Union) and provided by the USDA National Small Grain Collection.

All lines were planted at the Wenjiang Experimental Station (103°51′E, 30°43′N) of Sichuan Agricultural University in the 2017–2018 cropping season. The former crop was rice. To maintain consistent growth conditions, all the materials were planted within an area of ~ 200 m^2^. The experiments exploited two planting patterns, i.e., mono-cropping (MC) of *Ae. tauschii* and inter-cropping (IC) of wheat-*Ae. tauschii*. Each MC *Ae. tauschii* accession and each IC wheat-*Ae. tauschii* combination were planted in 5 × 5 rows with a row length of 2.0 m and row spacing of 30 cm. For the MC growth condition, five *Ae. tauschii* seeds were spaced and sown in each row (Fig. S1). For the IC growth condition, five *Ae. tauschii* seeds were inter-sown with 10 wheat seeds in each row in 2017. The sowing date was 4 November 2017.

### Phenotypic measurements

The measured traits at the seedling stage included plant height, leaf length and tiller number, and measurements were performed on 12 March 2018. The measured traits at the adult stage included plant height, flag leaf length, tiller number, spike length, number of spikelets, internode length, seed setting rate, heading time and flowering time. The plant height, leaf length, spike length, number of spikelets, internode length, and seed setting rate were the average values of the three highest tillers for each plant. All the measured plants were used to compare the difference in *Ae. tauschii* between MC and IC conditions. Significant differences were determined by Student’s t tests.

### RNA sequencing

On 12 March 2018, samples from four accessions under MC and IC were taken for RNA sequencing when the *Ae. tauschii* plants at vegetative periods were climbing for mono-cropping but erect for intercropping. For each treatment, 2–4 biological replicates were set. Whole plants harvested from the field were immediately snap-frozen in liquid nitrogen and then stored at -80 °C. Total RNA was extracted from the samples excluding roots using an RNAprep Pure Plant kit (TIANGEN, Beijing, China). Sequencing libraries were generated using the NEBNext® Ultra™ RNA Library Prep Kit for Illumina (New England Biolabs, USA). The libraries were sequenced using a HiSeq 2500 platform (Illumina, San Diego, CA, USA) following the standard protocol. RNA concentration and purity were measured using a NanoDrop 2000 (Thermo Fisher Scientific, Wilmington, DE). RNA integrity was assessed using the RNA Nano 6000 Assay Kit of the Agilent Bioanalyzer 2100 system (Agilent Technologies, CA, USA).

The clustering of the index-coded samples was performed on a cBot Cluster Generation System using TruSeq PE Cluster Kit v4-cBot-HS (Illumina). After cluster generation, the prepared libraries were sequenced on an Illumina platform, and paired-end reads were generated. Contaminated and low-quality reads were discarded by imposing a Q30 threshold of 90% and a maximum of 0.2% ambiguous base calls. Reads were mapped to the *Ae. tauschii* reference genome (https://www.ncbi.nlm.nih.gov/assembly/GCF_001957025.1) using HISAT2 with default settings for parameters [20]. FPKM (Fragments Per Kilobase of transcript per Million fragments mapped) was used to quantitatively estimate the value of gene expression [21].

### Differential expression analysis

Differential expression analysis was performed using the DESeq2 R package [22]. Differentially expressed genes (DEGs) of each accession under competitive stress were determined with a false discovery rate (FDR) threshold < 0.05 and |log2FC| ≥ 1 (FC means fold change). The gene with an FPKM value of 0 was given a value close to 0 to calculate the fold change.

To discover common DEGs shared by all the analysed accessions under inter-cropping conditions, K-means analysis was performed. The k value was set to 30 according to the gene expression patterns.

### GO and KEGG enrichment

Gene Ontology (GO) enrichment analysis of DEGs was implemented by GOseq, which is an R package based on the Wallenius noncentral hypergeometric distribution [23]. KOBAS software was used to test the statistical enrichment of target genes in KEGG pathways [24]. GO categories and KEGG pathways with P values ≤ 0.05 were defined as significantly enriched.

### Weighted gene co-expression network analysis (WGCNA)

Co-expression networks were built using weighted gene co-expression network analysis (WGCNA) in BMKCloud (www.biocloud.net) [25]. The parameters used in the WGCNA were as follows: FPKM ≥ 1; cv (Variation of FPKM) ≥ 0.5; hierarchal clustering tree: dynamic hybrid tree cut algorithm; power: 13; minimum module size: 30; minimum height for merging modules: 0.31455. The candidate co-expression network was visualized by Cytoscape (version 3.4.0, released on May 13, 2016). In the co-expression network, the 10 genes with the highest degree of connectivity were regarded as hub genes. In the net, circular nodes represent genes, and edges represent connections.

### Quantitative accuracy analysis of RNA-seq

Eighteen genes were randomly selected and verified by quantitative real-time PCR (qRT-PCR). The TaKaRa Prime Script TMRT Reagent Kit with gDNA Eraser (Perfect Real Time; TaKaRa, Shiga, Japan) was used to synthesize cDNA according to the manufacturer’s instructions. qRT-PCR was performed using SYBR Premix Ex Taq™ II (TaKaRa, Shiga, Japan). The 20 µl mixtures for PCRs consisted of 10 µl of 2× SYBR Green II Mix, 0.4 µl of each forward and reverse primer, 2 µl of cDNA, and 7.2 µl of ddH2O. The PCR program was 94 °C for 5 min, followed by 35 cycles of 94 °C for 30 s, 58 °C for 30 s, and 72 °C for 30 s. Three biological replicates were conducted for each sample. The relative expression levels were calculated using the 2^−ΔΔC^t method [26]. Specific primers for qRT-PCR were designed using Primer 5.0 software (Table S1). GAPDH was used as a reference gene [27].

## Results

### Growth in response to shading stress

**Fig. 1:**
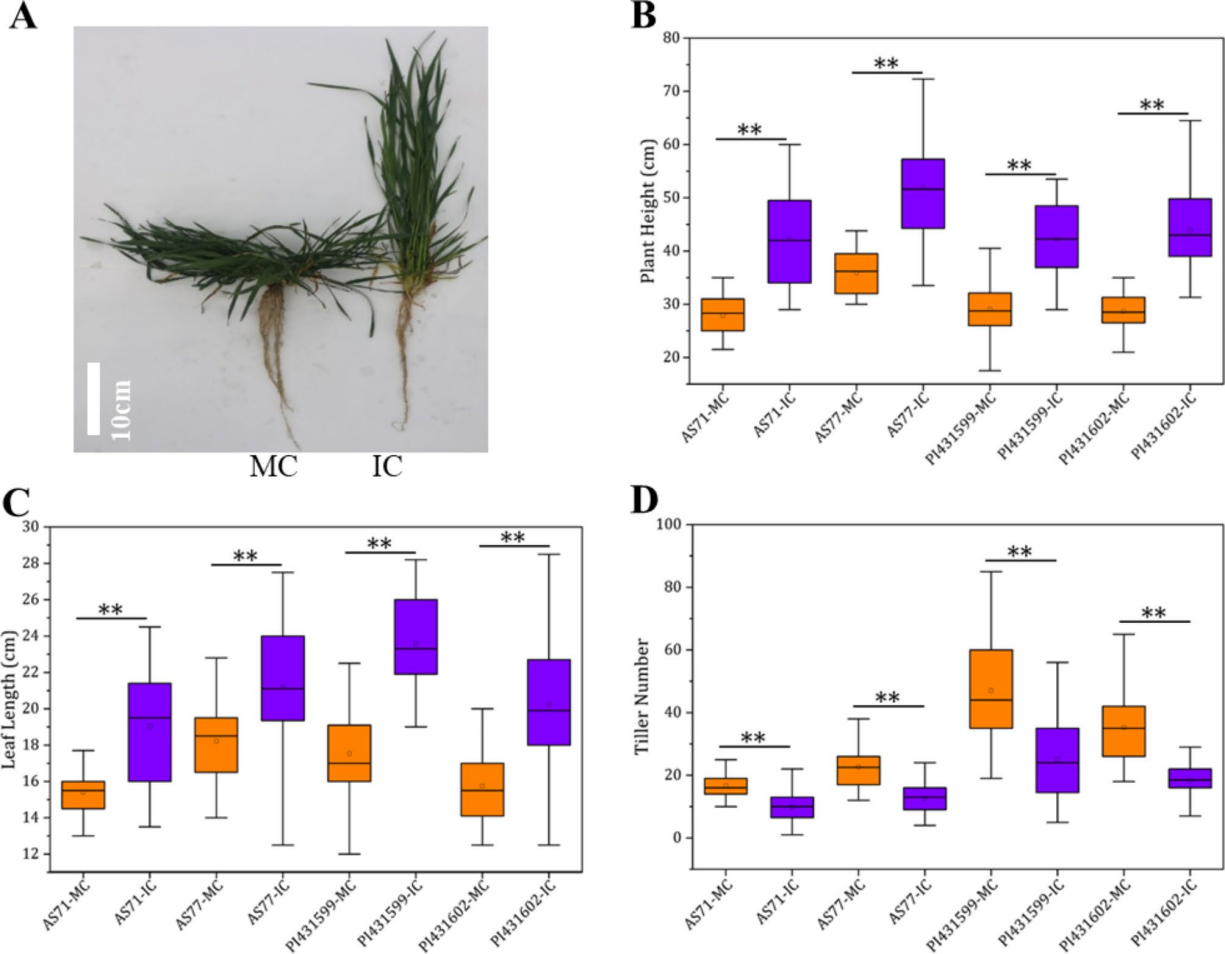
Trait comparison of four *Ae. tauschii* accessions between mono-cropping (MC) and inter-cropping (IC) at the seedling stage. **A** Plant morphology. **B** Plant height. **C** Leaf length. **D** Tiller number. ** Significance at the 0.01 probability level, * Significance at the 0.05 probability level

Four *Ae. tauschii* accessions belonging to subspecies *tauschii* (AS71 and AS77) and subspecies *strangulata* (PI431599 and PI431602) were used in this study. They were planted in an experimental field under mono-cropping (MC) and inter-cropping (IC) patterns (Fig. S1). Under IC, the plants of *Ae. tauschii* at the seedling stage were lower than those of the surrounding wheat plants, resulting in shading stress. The four analysed *Ae. tauschii* accessions in IC exhibited similar responses, including a more erect position of plants and elongated leaves but accompanied by reduced root growth and fewer tillers compared to MC (Fig. 1). In IC, the average height of the four materials increased by 14.7 cm, and the average leaf length increased by 4.2 cm. This indicated that the shading stress from wheat strongly changed the growth of *Ae. tauschii* seedlings regardless of genotype or taxon.

**Fig. 2:**
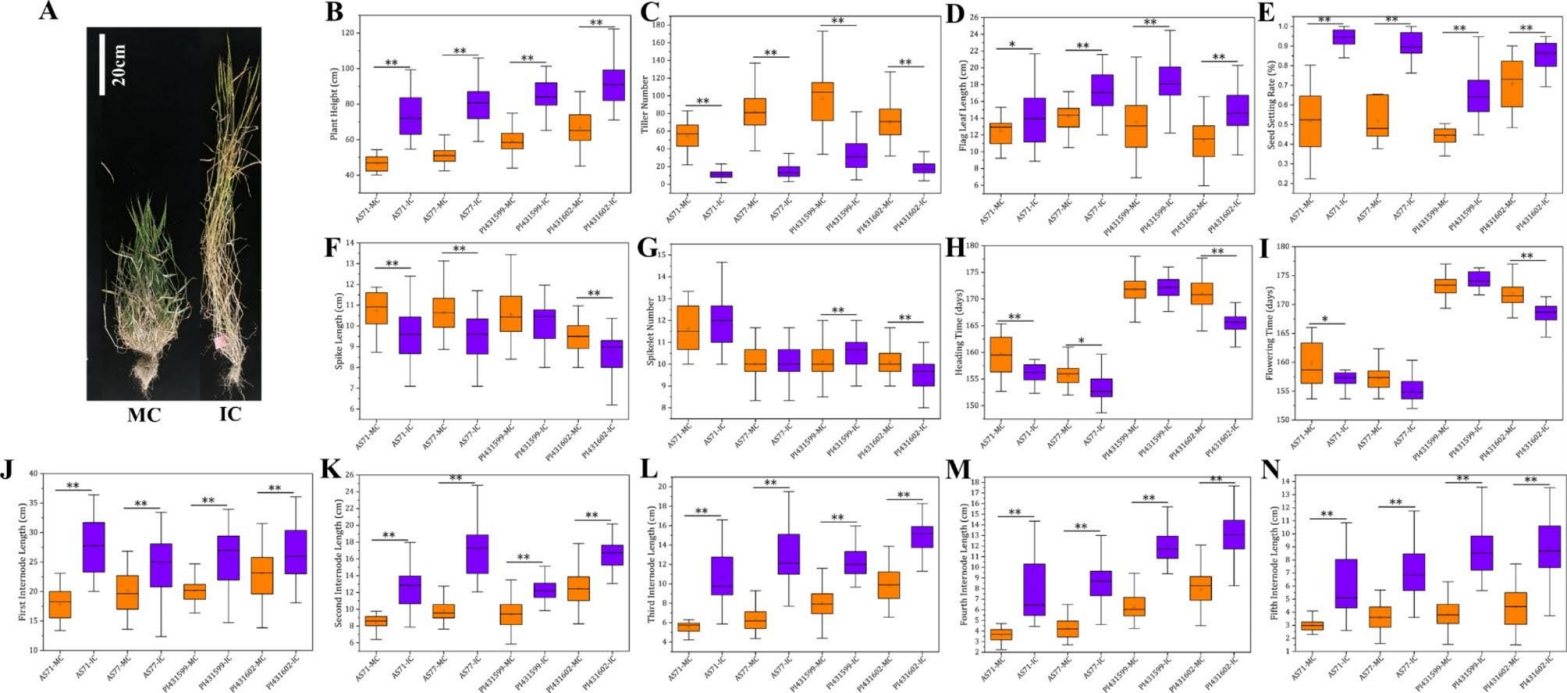
Trait comparison of four *Ae. tauschii* accessions between mono-cropping (MC) and inter-cropping (IC) at the adult stage. **A** Plant morphology. **B** Plant height. **C** Tiller number. **D** Flag leaf length. **E** Seed setting rate. **F** Spike length. **G** Spikelet number. **H** Heading time. **I** Flowering time. **J** First internode length. **K** Second internode length. **L** Third internode length. **M** Fourth internode length. **N** Fifth internode length. ** Significance at the 0.01 probability level, * Significance at the 0.05 probability level

We also compared the phenotypic changes of plants between MC and IC at the adult stage. The four *Ae. tauschii* accessions in IC all exhibited increased plant height, flag leaf length and seed setting rate but fewer tillers and roots (Fig. 2). For instance, the number of tiller changes was very evident (MC vs. IC: 54 vs. 11 for AS71, 82 vs. 15 for AS77, 98 vs. 33 for PI431599, 71 vs. 18 for PI431602). The average height of the four materials increased 26.4 cm, with subspecies *tauschii* increasing 28.3 cm and subspecies *strangulata* increasing 24.5 cm. The average length of internodes increased 5.9 cm, 4.6 cm, 5.2 cm, 4.9 cm and 3.8 cm from the first to the fifth internodes. The increase in plant height was actually caused by the increase in internode length. Furthermore, the average increase in the seed setting rate of the four materials was 27%. However, the phenotypic changes in other traits, including heading time, flowering time, spike length and number of spikelets, were dependent on *Ae. tauschii* accessions (Fig. 2). Among them, the average heading time was approximately 3 days earlier and the average flowering time was approximately 2 days earlier for the four materials under IC.

### Transcriptome analysis

**Fig. 3:**
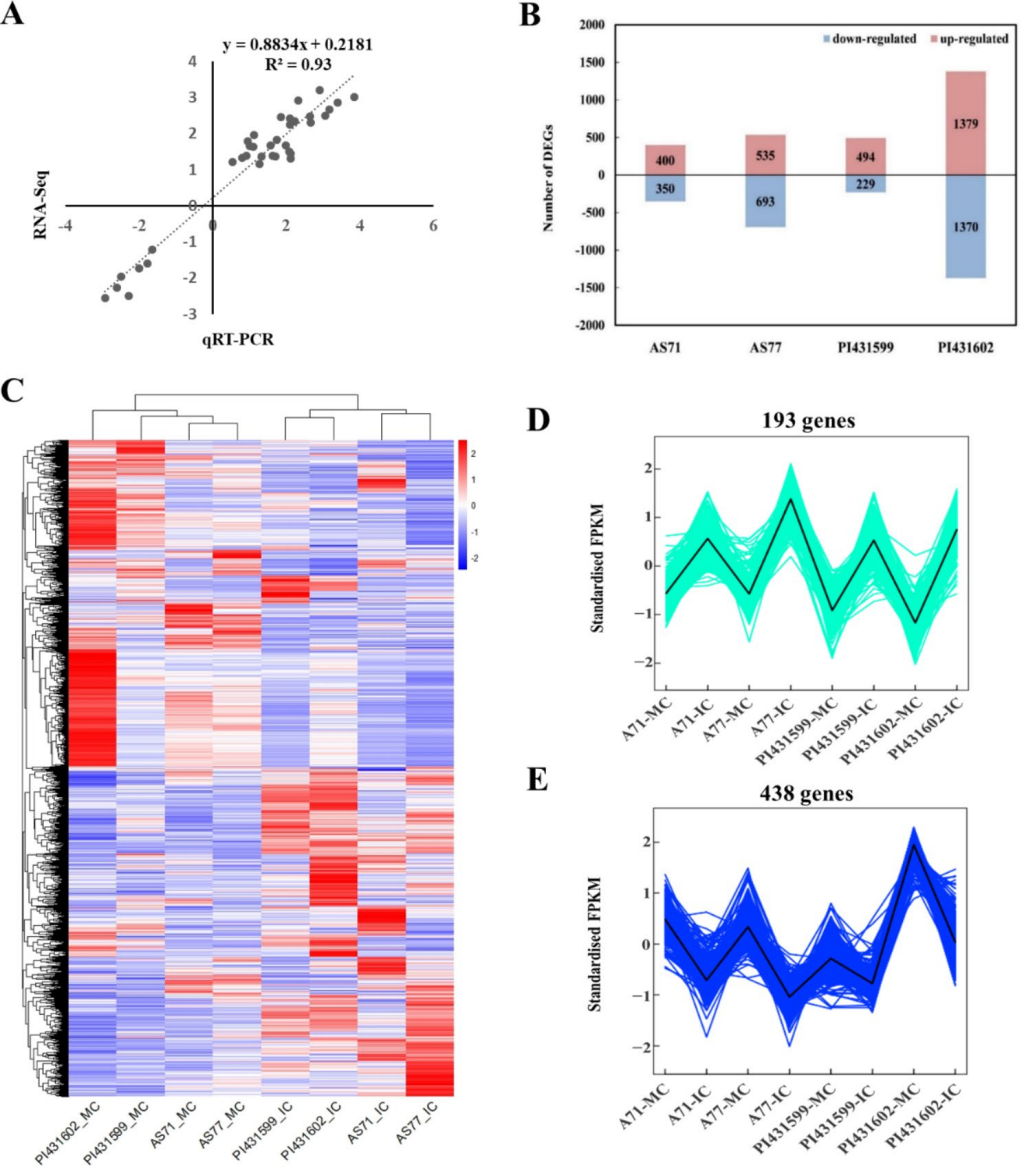
Gene expression under competitive stress. **A** Correlation analysis of the expression levels estimated using qRT-PCR and RNA-Seq. **B** The number of up- and downregulated differentially expressed genes (DEGs) in four accessions. **C** Expression profiles of 4294 DEGs. **D** Expression profiles of the common upregulated response genes. **E** Expression profiles of the common downregulated response genes

To decipher the gene expression responses under shading stress, we completed RNA-Seq analysis of *Ae. tauschii* at vegetative periods when plant morphology was distinct between MC and IC (Fig. 1A). RNA-Seq analysis was applied to 23 RNA samples, mostly showing high correlation coefficients between biological replicates (Fig. S2). To further test the accuracy of RNA-seq quantification, we randomly selected 18 expressed genes for qRT-PCR analysis. The linear regression of the expression level using the data from qRT-PCR and RNA-seq was then analysed. The R square was 0.93 (Fig. 3A), inferring that the results of RNA-seq quantitative analysis are trustworthy. On average, the Q30 base percentage was 93.92% or above. The clean reads of each sample were sequenced with the specified reference genome, and the alignment efficiency ranged from 79.75 to 94.64%. Most (83.13%) of the reads were uniquely mapped (Table S2).

### Gene expression under growth shading stress

To find *Ae. tauschii* genes that responded to shading stress caused by wheat in the seedling stage, we first analysed the DEGs between MC and IC. There were 4294 DEGs (Table S3), and the number was different among the four *Ae. tauschii* accessions varied from 723 in PI431599 to 2,749 in PI431602 (Fig. 3B). To detect the expression profiles of DEGs, we first carried out a simple cluster analysis of DEGs, found that it was difficult to find the common response genes (Fig. 3C), and then performed K-means analysis. Considering that the four accessions exhibited obvious shade-avoidance responses to light competition, this analysis focused on the common response genes, which were shared by the four *Ae. tauschii* accessions and either upregulated or downregulated. There were 631 common response genes (Table S4). Of these, 193 had higher expression levels in IC than in MC (Fig. 3D). However, many more genes (438) were downregulated in IC (Fig. 3E).

### Functional enrichment analysis of common DEGs under growth shading stress

**Fig. 4:**
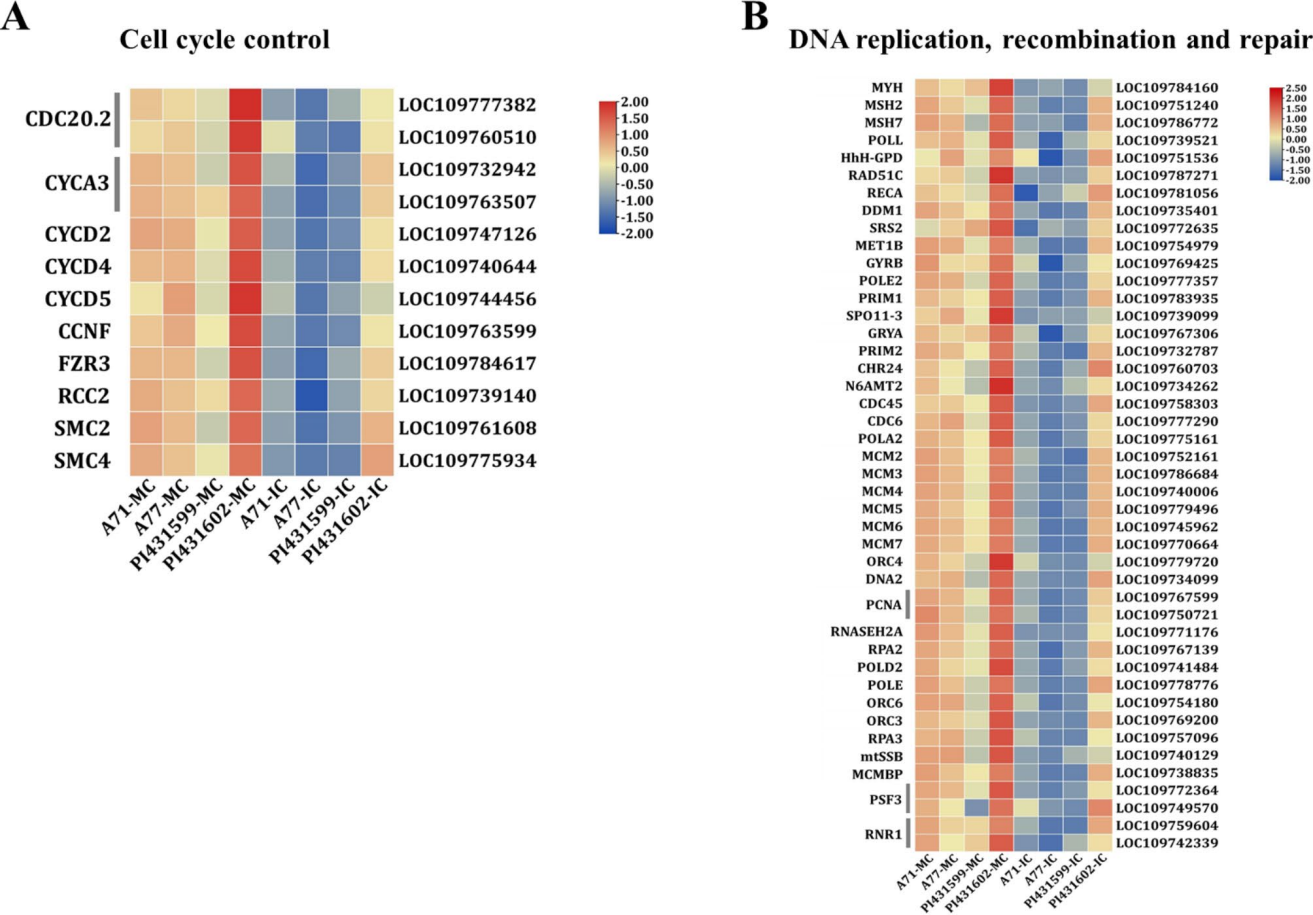
Expression level of the top enriched pathways in the 438 common downregulated response genes. **A** Genes involved in cell cycle control. **B** Genes involved in DNA replication, recombination and repair. The colors of heatmap vary from blue to red by normalizing the log_2_ (FPKM) of each gene

To gain insight into the functional categories of the 631 common response genes, we performed Gene Ontology (Table 1) and KEGG enrichment analyses (Table 2). Cell cycle-, DNA replication- and plant hormone signal transduction-related genes were clearly enriched among the downregulated genes in IC. Unexpectedly, all 12 common response genes for cell cycle control were downregulated, including two *cell division cycle 20.2* (*CDC20.2*), six *cyclins* (2 *CYCA3* and *CYCD2*, *CYCD4*, *CYCD5*, *CCNF*), protein *fizzy-related 3* (*FZR3*), *regulator of chromosome condensation* (*RCC2*), and two *structural maintenance of chromosomes* (*SMCs*) (Fig. 4A, Table S5). Additionally, the 44 common response genes for DNA replication, recombination and repair were all downregulated (Fig. 4B, Table S5). Of these, 32 were involved in DNA replication, such as six *DNA replication licensing factor MCM* (*MCM2*-*MCM7*), *cell division control proteins* (*CDC6*, *CDC45*), two *proliferating cell nuclear antigens* (*PCNA*), two *DNA replication complex GINS proteins PSF3*, two *ribonucleoside-diphosphate reductase large subunits* (*RNR1*) and *DNA helicases* (*DDM1*, *SRS2*); seven were involved in DNA recombination, including *DNA repair proteins* (*RAD51C* and *RECA*), *DNA polymerase epsilon subunit B* (*POLE2*), *protein-lysine N-methyltransferase n6amt2* (*N6AMT2*), *DNA (cytosine-5)-methyltransferase 1B* (*MET1B*), *chromatin remodelling 24-like* (*CHR24*), and *DNA topoisomerase 6 subunit A3* (*SPO11-3*); and five were involved in DNA repair, including *adenine DNA glycosylase* (*MYH*), *DNA polymerase lambda* (*POLL*), *DNA glycosylase* (*HhH-GPD*), and *DNA mismatch repair protein MSH* (*MSH2*, *MSH7*).

**Table 1:** Main enriched GO categories in the 631 common response genes

Expression pattern	ID	Description	Q value
Up-regulated genes	GO:0004197	cysteine-type endopeptidase activity	0.001581
	GO:0005615	extracellular space	0.003188
	GO:0005764	lysosome	0.003842
	GO:0004674	protein serine/threonine kinase activity	0.013115
	GO:0009514	glyoxysome	0.017737
Down-regulated genes	GO:0006270	DNA replication initiation	9.17E-14
	GO:0006334	nucleosome assembly	8.2E-11
	GO:0006260	DNA replication	3.26E-08
	GO:0007049	cell cycle	1.05E-06
	GO:0006342	chromatin silencing	5.13E-05
	GO:0032508	DNA duplex unwinding	0.000245
	GO:0071897	DNA biosynthetic process	0.003908
	GO:0007018	microtubule-based movement	0.009109
	GO:0006268	DNA unwinding involved in DNA replication	0.011548
	GO:0048366	leaf development	0.028369
	GO:0007076	mitotic chromosome condensation	0.031739
	GO:0009886	post-embryonic animal morphogenesis	0.047169
	GO:0045740	positive regulation of DNA replication	0.047882
	GO:0034644	cellular response to UV	0.047882

The Q value is the P value of the enrichment corrected with the Bonferroni method for multiple testing

**Table 2:** Main enriched Go categories and KEGG pathway in the 631 common response genes

Expression pattern	ID	Description	Q value
Up-regulated genes	ko00630	glyoxylate and dicarboxylate metabolism	1.05E-06
	ko00232	caffeine metabolism	5.87E-05
	ko00620	pyruvate metabolism	0.001277
	ko00220	arginine biosynthesis	0.001848
	ko01200	carbon metabolism	0.002738
	ko00410	beta-Alanine metabolism	0.004164
	ko00910	nitrogen metabolism	0.004964
	ko01230	biosynthesis of amino acids	0.008801
	ko00280	valine, leucine and isoleucine degradation	0.018952
	ko00310	lysine degradation	0.018952
	ko00770	pantothenate and CoA biosynthesis	0.026426
	ko00071	fatty acid degradation	0.028025
	ko00380	tryptophan metabolism	0.033005
	ko00360	phenylalanine metabolism	0.036468
	ko01210	2-Oxocarboxylic acid metabolism	0.040942
	ko00561	glycerolipid metabolism	0.042773
	ko00592	alpha-Linolenic acid metabolism	0.043698
Down-regulated genes	ko03030	DNA replication	6.91E-21
	ko03410	base excision repair	3.82E-09
	ko00240	pyrimidine metabolism	6.42E-07
	ko03430	mismatch repair	3.14E-06
	ko03420	nucleotide excision repair	1.51E-05
	ko00230	purine metabolism	1.67E-05
	ko03008	ribosome biogenesis in eukaryotes	0.000124
	ko03440	homologous recombination	0.000218
	ko03010	ribosome	0.011873
	ko03450	non-homologous end-joining	0.014704
	ko04075	plant hormone signal transduction	0.039937

The Q value is the P value of the enrichment corrected with the Bonferroni method for multiple testing

**Fig. 5:**
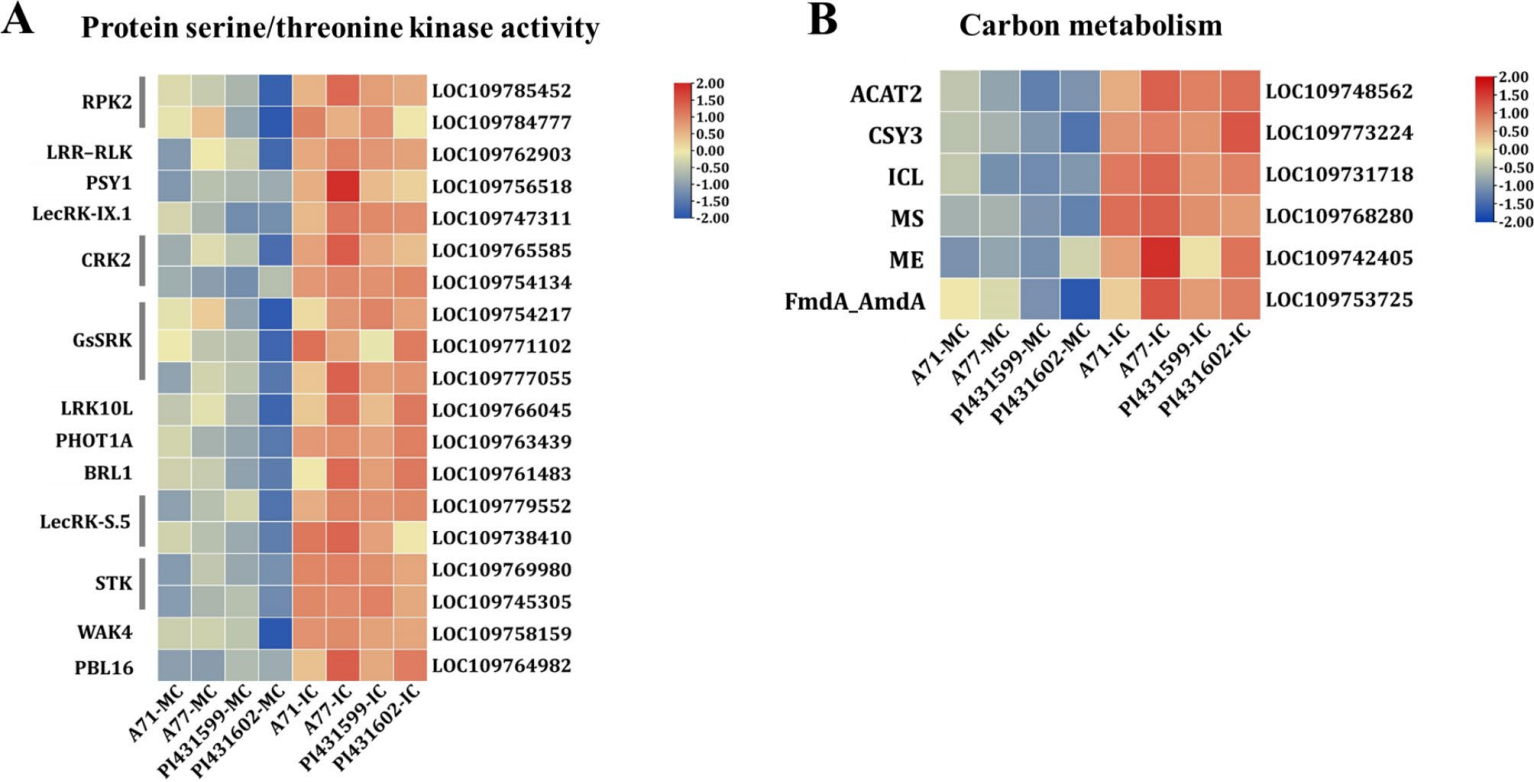
Expression level of the top enriched pathways in the 193 common upregulated response genes. **A** Genes involved in protein serine/threonine kinase activity. **B** Genes involved in carbon metabolism. The colors of heatmap vary from blue to red by normalizing the log_2_ (FPKM) of each gene

The upregulated genes under IC were mainly enriched in protein serine/threonine kinase activity, carbon metabolism, and cysteine-type endopeptidase activity. Unexpectedly, all 19 common response genes involved in the protein serine/threonine kinase activity pathway were upregulated (Fig. 5A, Table S5). Most of them (16) belonged to the receptor-like kinase (RLK/Pelle) protein kinase family, including three *G-type lectin S-receptor-like serine/threonine-protein kinases* (*GsSRK*), two *cysteine-rich receptor-like protein kinases 2* (*CRK2*), three *LRR receptor-like serine/threonine-protein kinases* (*RPK2*, *LRR–RLK*), two probable *L-type lectin-domain containing receptor kinases S.5* (LecRK-S.5) and *wall-associated receptor kinase 4-like* (*WAK4*), probable *serine/threonine-protein kinase PBL16*, probable *leucine-rich repeat receptor-like protein kinase* (*BRL1*), *leaf rust 10 disease-resistance locus receptor-like protein kinase* (*LRK10L*), *L-type lectin-domain containing receptor kinase IX.1-like* (*LecRK-IX.1*), and *tyrosine-sulfated glycopeptide receptor 1-like* (*PSY1*). In addition, 2 were probable *serine/threonine protein kinases (STKs*) belonging to the cyclin-dependent kinase (CDK) kinase family, and one is *phototropin-1a isoform x1* (*PHOT1A*) belonging to the AGC kinase family. Additionally, six genes associated with the carbon metabolism pathway were all upregulated (Fig. 5B, Table S5). *Acetyl-CoA acetyltransferase* (*ACAT2*) is mainly involved in lipid metabolism. The other five were involved in energy production and conversion, including *citrate synthetase 3* (*CSY3*), *isocitrate lyase* (*ICL*), *malate synthetase* (*MS*), *NADP-dependent malic enzyme* (*ME*) and the *acetylase/formamide family* (*FmdA_AmdA*). Additionally, we conducted a specific analysis of 13 genes associated with crucial pathways using qRT-PCR. The results revealed that genes implicated in the cell cycle and DNA replication pathways (CDC20.2, CYCA3;2, CYCD5;3, MSH2, CDC45, MCM3) exhibited downregulation, while genes related to kinase activity and carbon metabolism pathways (RPK2, BRL1, WAK4 ACAT2, MS, ME) showed upregulation (Fig. S3). These findings were consistent with the transcriptome results.

### Co-expression network

**Fig. 6:**
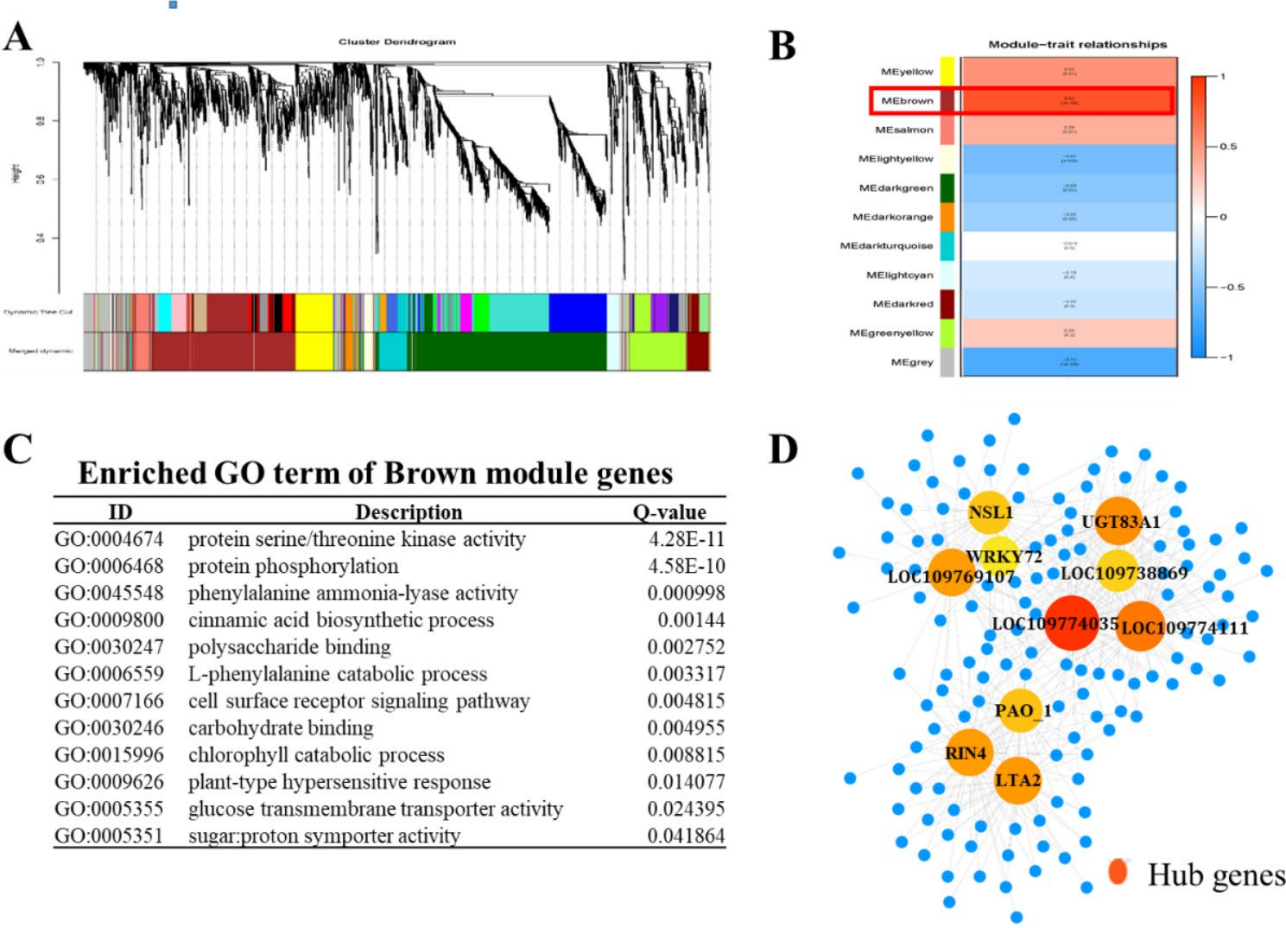
WGCNA of differentially expressed genes. **A** Hierarchical cluster tree showing co-xpression modules identified by WGCNA. Each leaf in the tree is one gene. The major tree branches constitute 11 modules labelled by different colours. **B** Module-sample association. Each row corresponds to a module. The name of modules is indicated on the left. Each column corresponds to a specific sample. The colour of each cell at the row-column intersection indicates the correlation coefficient between the module and sample. A high degree of correlation between a specific module and sample is indicated by red. **C** The top enriched GO categories in brown module genes. **D** Co-expression network in the brown module with a KME higher than 0.9 visualized by Cytoscape

To identify vital genes of *Ae. tauschii* specific to shading stress, the 4294 DEGs were used for co-expression network analysis using WGCNA (Fig. 6A). This analysis revealed 11 modules, i.e., 11 highly connected gene clusters. The brown module was positively correlated with the shading response (Fig. 6B, Table S6). GO analysis indicated that they were enriched in protein serine/threonine kinase activity, protein phosphorylation, cinnamic acid biosynthetic process, chlorophyll catabolic process and phenylalanine ammonia-lyase activity (Fig. 6C). The 10 genes with the highest number of nodes were regarded as hub genes (Fig. 6D, Table S7). The gene with the most nodes encoded a protein of unknown function (LOC109774035, 66 nodes). A gene encoding WRKY transcription factor 72 (LOC109776904, 24 nodes) was among the top 10 hub genes as well as in shading response common genes. There were 71 subgenes under *WRKY72* node, including 11 protein kinases, which were 2 *serine/threonine-protein kinase RIPK*, 2 *wall-associated receptor kinase 2* (*WAK2*), 2 *cysteine-rich receptor-like protein kinase* (*CRK10* and *CRK6*), 2 *receptor-like protein kinase FERONIA*, 2 *L-type lectin-domain containing receptor kinase* (*LecRK-IX.1* and *LecRK-IV.1*) and *Rust resistance kinase Lr10* (*LRK10*). Another hub gene, *UDP glycosyltransferase 83A1* (LOC109776791, 47 nodes), had 47 subgenes in its network, including 8 protein kinases, all of which were the same as those in the *WRKY72* network. Additionally, we performed qRT-PCR analysis on the top three hub genes and *WRKY72*. The results showed that all of them exhibited up-regulation in expression under shade stress, which was in line with the transcriptome findings (Fig. S3).

## Discussion

Crops and weeds unavoidably face competition for resources such as sunlight. How low light stress caused by the shading of adjacent plants affects plant growth is not only a fundamental scientific question but is also of vital importance for agriculture, such as in weed control and crop breeding. The monocot *Ae. tauschii* is the D-genome donor of bread wheat [8], but it is also a weed found wheat fields. The present study indicated that shade avoidance is a strategy of *Ae. tauschii* plants to respond to low light stress. Shade-avoidance responses are associated with phenotypic changes that favour plants to obtain more light resources, which are called shade-avoidance syndromes [19, 28, 29]. *Ae. tauschii* altered growth in specific organs to avoid the shading of surrounding wheat plants in the field, such as forming a more erect position of plants and increasing stem and leaf length. However, these changes were accompanied by sacrificing the growth of other parts of the plants, such as fewer tillers and roots. The obvious reverse effects of shading on plants, i.e., growth increases in some parts of a plant but decreases in other parts of the plant were also observed in other plant systems, such as the dicot *Arabidopsis* [29, 30]. It is interesting to understand how the dual responses of shaded plants are harmonized. Intuitively, the growth increase and decrease should be controlled by independent strategies by which plants respond to low light stress.

A decrease in plant growth is a typical response to abiotic stresses such as severe drought and salt. The trade-off between growth and stress resistance is usually explained by energy/carbon limitations, since plants under stress divert substantial resources away from growth and towards a stress response [31, 32]. However, increasing evidence indicates that the trade-off mainly results from the active suppression of growth by stress signalling pathways [33–36]. Plant growth depends on cell growth, which is the process by which cells accumulate mass by cell division and increase in physical size by cell enlargement, and abiotic stress often impedes plant growth by repressing both cell division and cell expansion [37]. Here, an obvious transcriptomic response to all the analysed *Ae. tauschii* accessions under shading stress was that fewer genes were upregulated (193) than downregulated (438). Interestingly, ~ 15% (66) of downregulated genes were enriched in the regulation of cell division, including 12 for cell cycle regulation and 44 for DNA replication, recombination and repair. Of these, 12 were *cyclins* (2 *CYCA3* and *CYCD2*, *CYCD4*, *CYCD5*, *CCNF*) and *DNA replication licensing factors* (*MCM2*-*MCM7*). Cyclins are key molecular drivers of the cell cycle, and their downregulation inhibits cell division under drought and salt stresses [37]. MCMs play important roles in DNA replication, and their downregulation is associated with abiotic resistance [38–40]. Cell division suppression could result in a reduction in the number of specific organs, such as tillers and roots, of *Ae. tauschii*. In addition to the downregulation of genes directly involved in cell division, some upstream genes involved in the negative regulation of cell division were also identified. For instance, the receptor-like kinase *RPK2* is upregulated under shading stress (Fig. 5A). It has been confirmed to be involved in the maintenance of the root apical meristem by controlling cell proliferation and affecting meristem size [41]. The roots of *RPK2*-overexpressing transgenic lines were diminished compared with those of the wild type [42]. The results suggested that shading stress signals may systematically activate gene systems to inhibit growth.

Although cell division and cell enlargement frequently go together, in some cases growth may be due mostly to cell enlargement. For instance, the gibberellin (GA)-induced growth of lettuce hypocotyls is primarily due to cell elongation [43]. Cell elongation could be exploited to explain the growth elongation of specific organs, such as stem and leaf elongation, which is the strategy of *Ae. tauschii* plants to obtain more light resources under shading stress. Consistent with this presumption, 16 out of 19 upregulated *Ae. tauschii* genes enriched in protein serine/threonine kinase activity under shading conditions belonged to RLK/Pelle family. RLK/Pelle proteins have been implicated in the mechanical properties of the cell wall, which is vital for cell expansion [44]. For instance, wall-associated kinases (WAKs) are a subclass of RLKs and are linked to cell elongation [45, 46]. The upregulation of WAK-like kinase 4 gene expression was associated with stress responses, such as to salt [47, 48]. In addition, *BRL1* (*BRASSINOSTEROID RECEPTOR-LIKE1*) is the main receptor of the brassinosteroid hormone and is expressed in vascular tissues and regulates shoot vascular development [49, 50]. Loss-of-function mutants *bri1* in *Arabidopsis* showed severe dwarfism; in contrast, overexpression of *BRL1* manifested as shoot elongation [51]. The upregulation of *BRL1* in *Ae. tauschii* under shading stress suggested that the brassinosteroid (BR) hormone may be involved in the formation of shade-avoidance syndromes.

Carbon metabolism is intimately linked to growth and stress responses, and tight control of their fluxes in cellular metabolism and throughout the plant is essential to ensure survival and reproduction under environmental constraints [52]. Abiotic stress affects energy supply. A study of shaded maize leaves found that the levels of amino acids, carbohydrates, lipids, nucleotides and related metabolites increased significantly under carbon starvation [53]. When stressed, to ensure an adequate energy supply, stressed plants respond to carbon metabolism, mainly by transferring a large amount of energy to stress resistance to prevent or repair stress-induced damage and maintain intracellular homeostasis [31, 32, 54, 55]. Five genes involved in energy production and conversion were upregulated in IC. In response to abiotic stress, plants divert substantial resources to resist stress to maintain cellular homeostasis. To ensure an adequate energy supply, stressed plants will generate a response to carbon consumption.

In the co-expression network, hub genes are expected to play an important role in shading stress. Of the 10 identified hub genes, the function of the top two genes (LOC109774035 and LOC109774111) in the connectivity ranking was unknown. Therefore, they are interesting candidates for further functional characterization. The third most interactive gene was *UDP glycosyltransferase 83A1* (*UGT83A1*, LOC109776791). *UGT83A1* glycosylates most of the lignin precursors and flavonoids, and its overexpressing lines showed strong abiotic stress tolerance [56], so it could potentially play key roles in coping with shading stress in *Ae. tauschii*. Another highly connected gene in the network was WRKY transcription factor (*WRKY72*, LOC109776904). WRKYs have multiple roles, including plant development, abiotic stress, hormone signalling, and primary and secondary metabolism [57–61]. WRKYs act as activators of the same ABA-inducible promoter and are related to the induction of abscisic acid/stress-related genes [62, 63]. During the shade-avoidance response, *WRKY26*, *45*, and *75* restrict root growth and development [64]. Since WRKY was regarded as both a hub gene and a common gene, it could play an important role in balancing growth and shading stress by reprogramming gene expression, such as cell cycle- and serine/threonine kinase-related genes.

## Conclusion

**Fig. 7:**
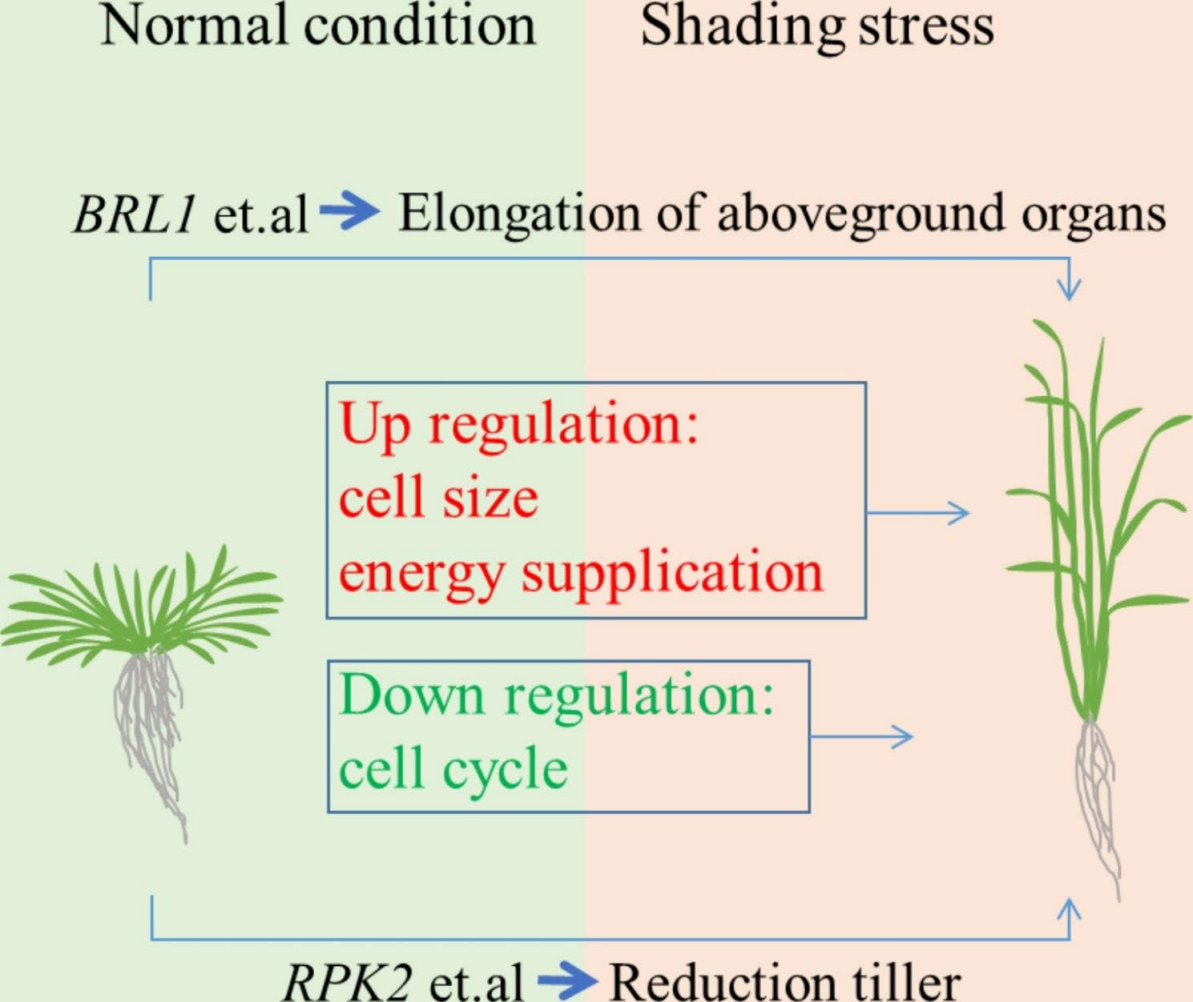
Relationship between stress signalling and plant growth. When the *Ae. tauschii* under shading stress with wheat, the response to light may mainly adopt two modes. On the one hand, to avoid the harsh environment of shade, plants grow taller and longer by increasing energy supply (carbon metabolism). On the other hand, stress defence is activated, and growth is inhibited. Abiotic stress usually hinders plant growth by inhibiting cell division and cell expansion, such as reducing tillers to a certain extent

The use of four *Ae. tauschii* accessions allowed for the detection of a robust set of genes that play a role in the shade-avoidance response. Defending against stress and actively inhibiting growth are two complementary strategies for plants to cope with adverse environments [35]. When the accessions of *Ae. tauschii* are under shading stress by wheat, the response to low light may mainly adopt two modes. On the one hand, to avoid the harsh environment of shade, plants grow taller and longer by increasing energy supply (carbon metabolism). On the other hand, stress defence is activated, and growth is inhibited. Abiotic stress usually hinders plant growth by inhibiting cell division and cell expansion, such as by reducing tillers to a certain extent (Fig. 7). This is an adaptive strategy to maximize survival. Since WRKY was regarded as both a hub gene and a common gene, it could be regarded as a candidate gene for further study. By introducing the hub gene into common wheat, the weed-like characteristics of *Ae. tauschii* can be incorporated, enabling the wheat plants to compete more effectively with weeds in harsh environments, leading to weed control. Due to experimental limitations, the aboveground tissues was taken for transcriptome sequencing in this experiment, and although the mechanism of shading stress specifically affecting stem and leaf tissues was not extensively explored, this study sheds new light on the gene expression changes and molecular processes involved in the response and avoidance of shading stress in the overall aboveground parts of *Ae. tauschii*, which may aid more effective development of shading stress avoidance or cultivars in wheat and other crops in the future.

## Electronic supplementary material

Below is the link to the electronic supplementary material.

Supplementary Material 1

Supplementary Material 2

Supplementary Material 3

Supplementary Material 4

Supplementary Material 5

Supplementary Material 6

Supplementary Material 7

Supplementary Material 8

## Data Availability

All data generated or analyzed during this study are included in this published article and its supplementary information files. The RNA-seq data generated by this study have been deposited in the National Center for Biotechnology Information Sequence Read Archive (accession no. PRJNA882581).
