# Supplementary material for: Transcriptomic analysis provides insight into the genetic regulation of shade avoidance in Aegilops tauschii
Source: BMC Plant Biol. 2023 Jun 23;23:336. doi: 10.1186/s12870-023-04348-y (PMC10288681; doi:10.1186/s12870-023-04348-y)

Supplementary material legends

**Fig. S1** Planting setup overview and examples of *Ae. tauschii* plants under inter-cropping (IC). **A** The experiments involved two planting patterns: mono-cropping (MC) of *Ae. tauschii* and inter-cropping (IC) of wheat with *Ae. tauschii*. **B** A seedling of *Ae. tauschii* shaded by wheat plants under IC (near the bottle). **C** An adult *Ae. tauschii* plant taller than the adjacent wheat plants under IC

Fig. S2 Pearson's correlation coefficient between biological replicates of different samples

Fig. S3 qRT-PCR analysis of some important genes

**Table S1** Primers used for validation of expressed genes by qRT-PCR

**Table S2** Summary of sequencing data quality

**Table S3** List of 4294 differentially expressed genes

**Table S4** List of differentially expressed genes in two k-means clusters

**Table S5** Top enriched pathways involving growth and stress responses in the 631 common response genes

**Table S6** List of differentially expressed genes in the Brown module

**Table S7** Top 10 hub genes in the Brown module based on node count

**Fig. S1**


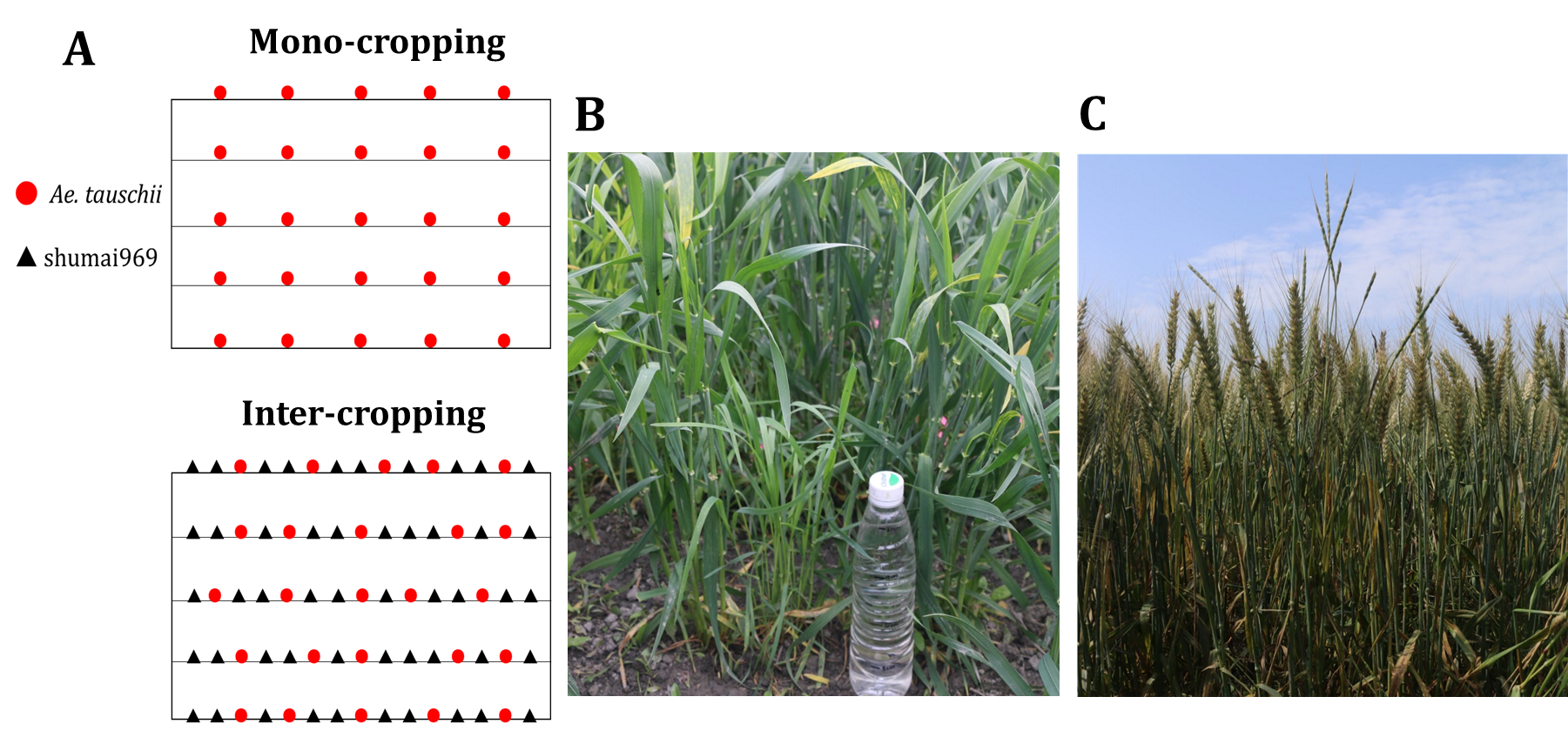


**Fig. S2**


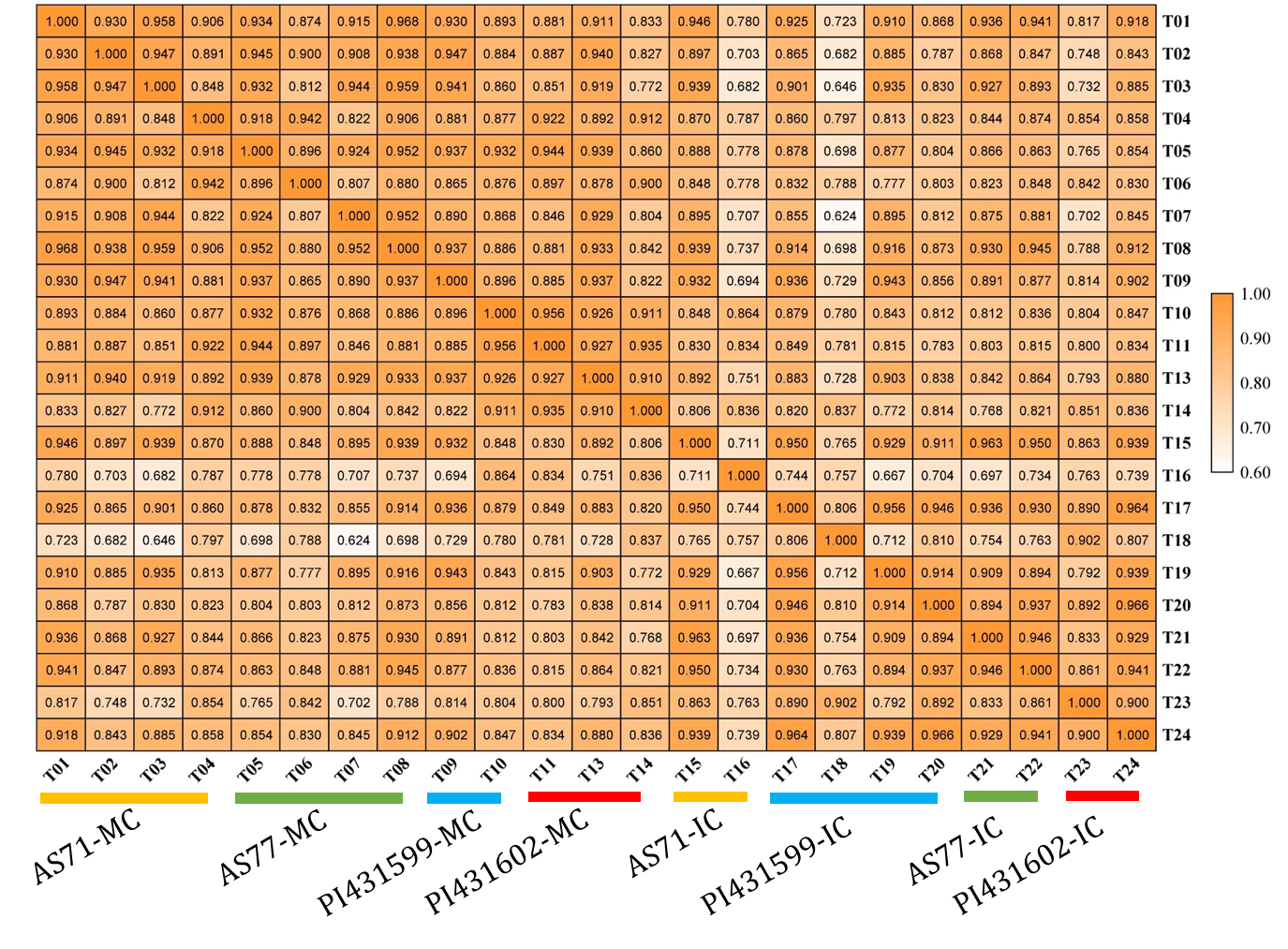


**Fig. S3**


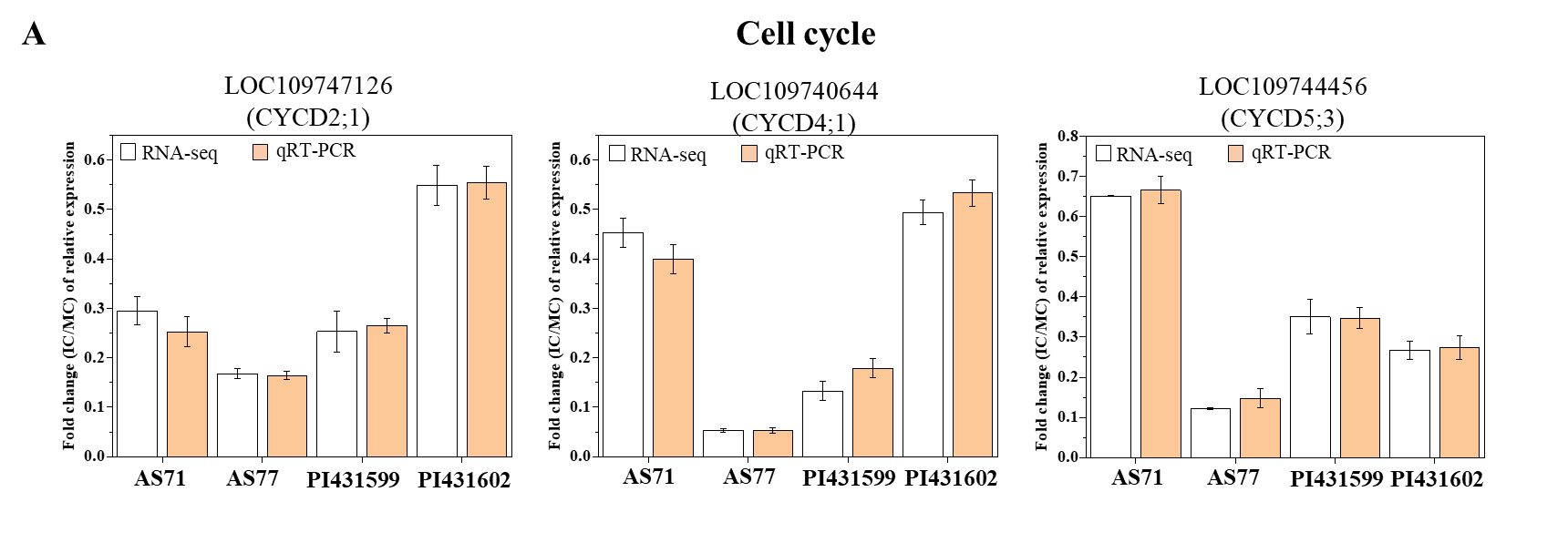


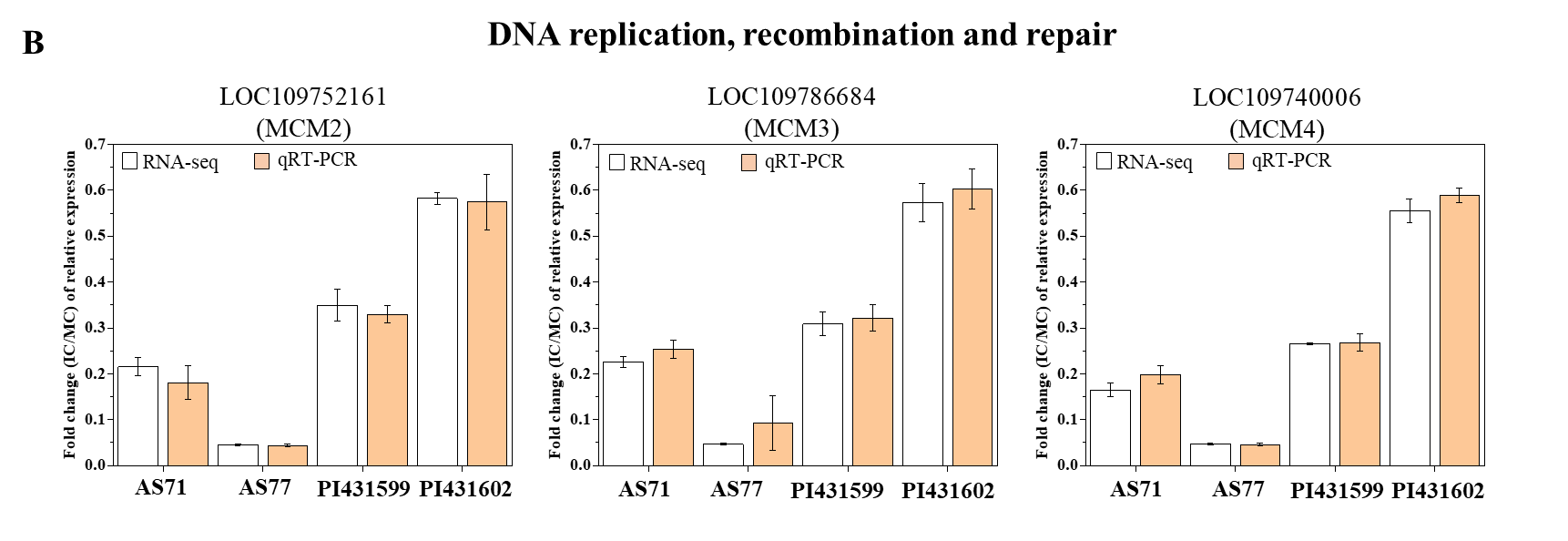


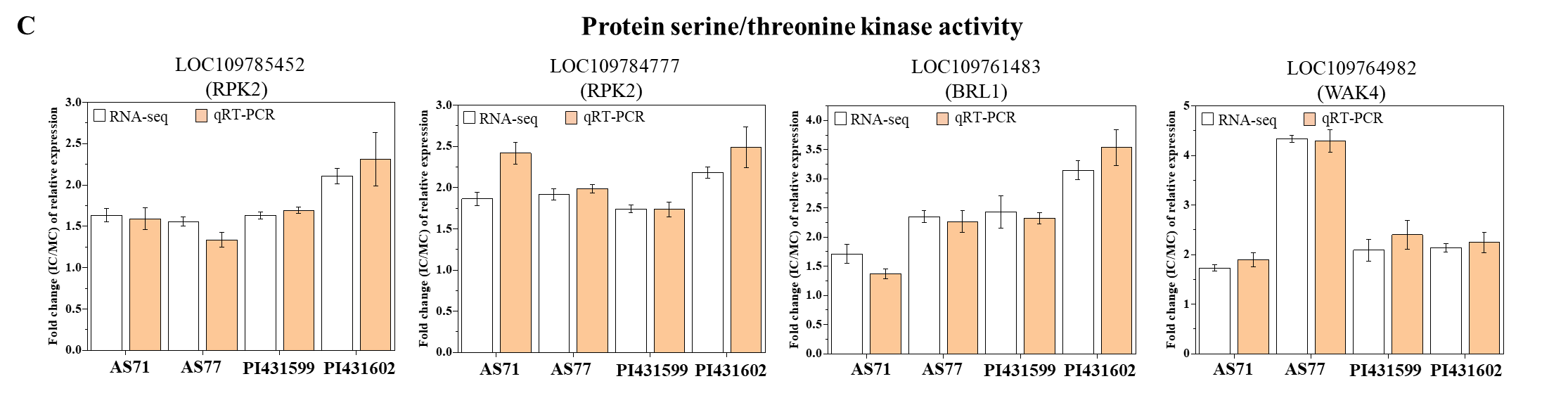


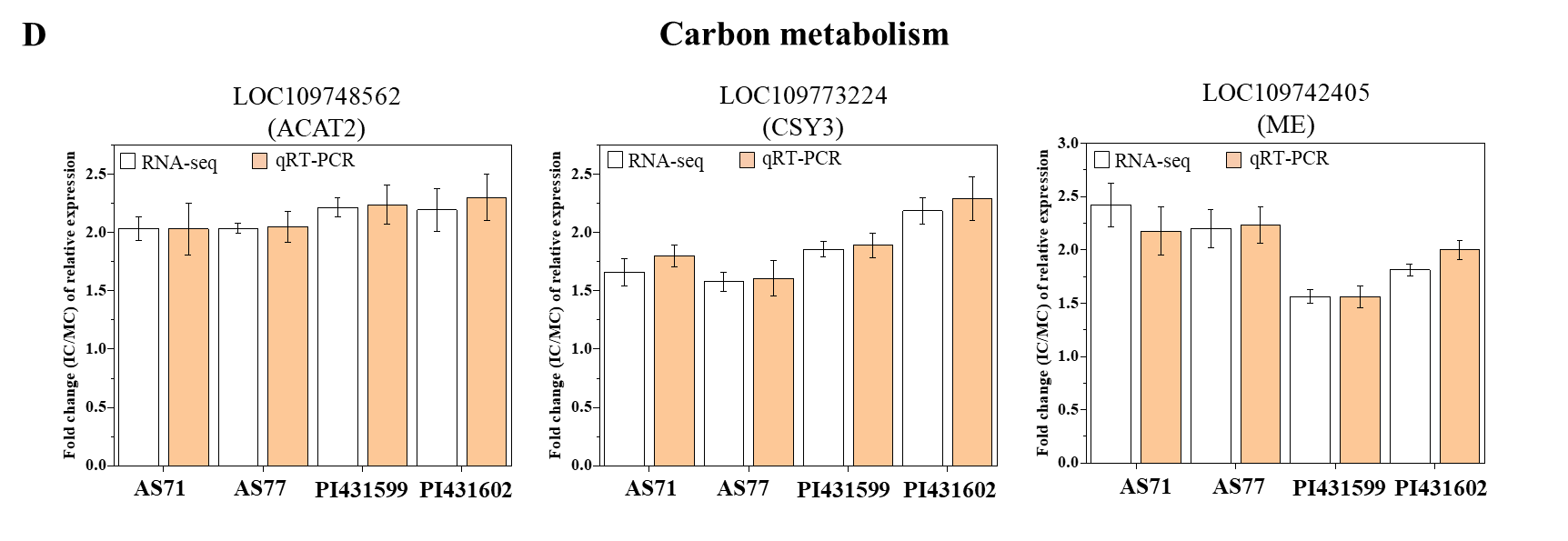


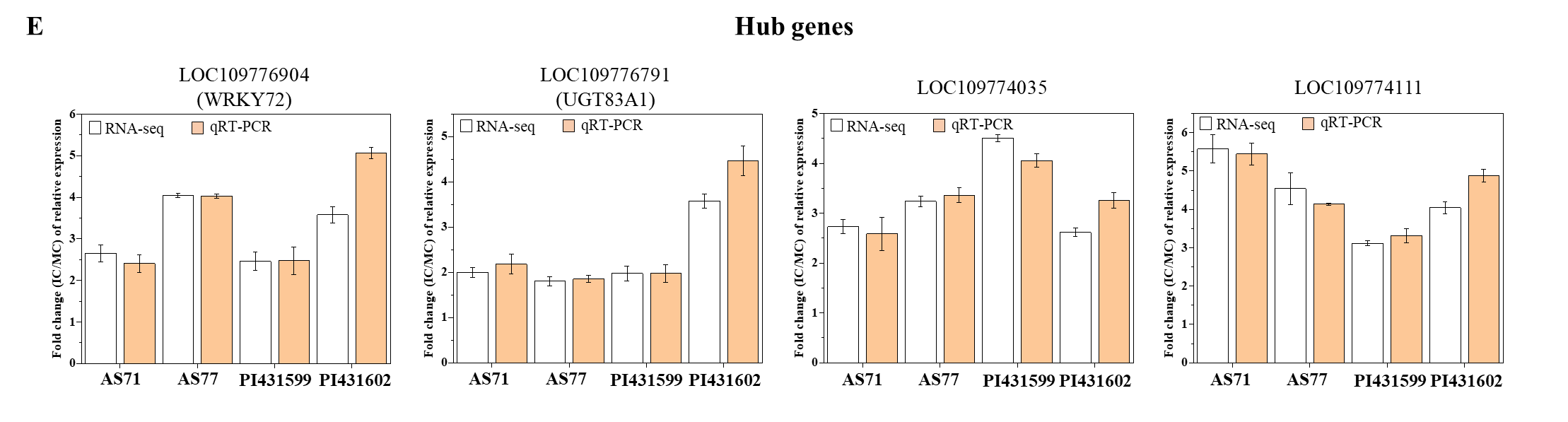

Supplement: Supplementary file 1 — Supplementary Material 1 [file 12870_2023_4348_MOESM1_ESM.docx]
